# “Assessment of an EMR-integrated Onco-Insight hospital-based cancer registry system for data completeness and data entry turnaround time at a tertiary care cancer center in India”

**DOI:** 10.3389/fonc.2026.1852225

**Published:** 2026-06-09

**Authors:** Amey Oak, Sandhya Cheulkar, Sivaranjini K., Riya John, Meghaj Jadhav, Dhanvi Patil, Esha Dashmukhe, Ashwini Patil, Sachin Wankhade, Pragati Gode, Aijimol Thakadiyil, Ganesh Balasubramanium, Gauravi Mishra, Rajesh Dikshit, Pankaj Chaturvedi, Sudeep Gupta

**Affiliations:** Division of Cancer Care, Hospital Cancer Registries & Survival Studies, Centre for Cancer Epidemiology (CCE), Advanced Center for Treatment, Research and Education in Cancer ACTREC, Tata Memorial Centre (TMC), Homi Bhabha National Institute (HBNI), Mumbai, India

**Keywords:** cancer registry, electronic medical records, India, software, data abstraction, data retrieval

## Abstract

**Background:**

Tata Memorial Centre maintains one of the largest hospital-based cancer registries (HBCR) in India. The registry captures demographic, diagnostic, and treatment information from the electronic medical record (EMR), while Patterns of Cancer Care and Survival Studies (POCSS) collect additional clinical variables including comorbidity, life style, family history of cancer, histopathology, and detailed treatment and follow-up information. As these data are distributed across multiple EMR modules, abstraction is time-consuming. In 2021, the HBCR software was upgraded to the Onco-Insight platform with EMR integration to facilitate automated data retrieval. This study evaluated the platform for data completeness and abstraction turnaround time.

**Methods:**

An observational evaluation using an intra-observer comparison design was conducted. Trained registry abstractors collected data using two approaches: conventional manual abstraction and the EMR-integrated Onco-Insight platform. The platform retrieves structured variables directly from the EMR while allowing manual entry for variables unavailable in structured format. Automated mapping from EMR modules to registry variables was implemented for sociodemographic, diagnostic, and treatment domains. Built-in validation rules, range checks, and mandatory field alerts were incorporated to enhance data quality. Data completeness was assessed for sociodemographic, diagnostic, and treatment variables and summarized as the proportion of cases with retrievable information. Data entry turnaround time was defined as the mean time required for abstraction and entry per case and assessed by cancer subsite for both HBCR and POCSS variables. Cases diagnosed and treated in 2021 were evaluated.

**Results:**

Of 37,495 registrations in 2021, 32,359 were confirmed cancer cases. Sociodemographic variables were retrieved for all cases (97.59%). Diagnostic variables were retrieved for 13,987 cases (43.22%), and treatment variables for 16,835 cases (50%), followed by manual validation. Manual review was mainly required for patients treated outside the institution, referred between TMC centers, or to distinguish initial therapy from treatment for recurrence or progression. Follow-up information after completion of cancer-directed therapy remained fully manual. Mean abstraction time decreased substantially for HBCR from 27.17 ± 4.40 minutes to 15.07 ± 3.00 minutes.

**Conclusion:**

The EMR-integrated Onco-Insight platform reduced abstraction time and improved efficiency while enabling unified entry for HBCR and POCSS. Further EMR standardization, expanded integration, and AI-enabled data extraction could strengthen registry efficiency and cancer surveillance.

## Introduction

Cancer remains a major public health challenge worldwide. According to International Agency for Research on Cancer estimates from GLOBOCAN 2020, there were approximately 19.3 million new cancer cases and 10.0 million cancer deaths globally, with projections indicating a substantial increase in burden over the coming decades. In India alone, cancer incidence and mortality continue to rise, with an estimated 1.4 million new cases and over 900,000 deaths (1, 2). In this context, high-quality cancer registry data are indispensable for clinical oncology, epidemiologic research, survival analysis, quality-of-care assessment and health system planning (3).

Quantifying the burden of cancer is central to informed public health decision-making and effective cancer control. Reliable estimates identify variations in cancer occurrence and outcomes across regions and population subgroups, guiding equitable resource allocation and targeted interventions (4, 5). Incidence, survival, and mortality remain the key epidemiological indicators for assessing cancer burden and evaluating the impact of cancer control strategies (3). Incidence reflects disease occurrence and prevention needs, survival indicates outcomes influenced by early detection and treatment, and mortality represents the overall population impact of cancer. Accurate interpretation of these measures requires methodological rigor, particularly when comparing populations or monitoring trends (3, 5).

High-quality cancer registry data supports clinical care, epidemiological research, survival analysis, and health system planning. The value of hospital-based cancer registries (HBCRs) depends on data completeness, validity, comparability, and timeliness, as outlined by the International Agency for Research on Cancer and the International Association of Cancer Registries (3, 5–7). Despite advances in electronic medical records (EMRs), registry abstraction often remains manual, making the process resource-intensive and susceptible to errors and delays, particularly in fragmented hospital information systems (8–10).

Tata Memorial Centre (TMC), one of India’s largest tertiary cancer institutions, has registered approximately 881,525 cancer cases since 1985 in its hospital-based cancer registry (HBCR), providing an important resource for epidemiological research, clinical audits, survival analysis, and health system planning. In 2021 alone, 37,495 cases were registered, with 32,359 confirmed cancers. The Patterns of Cancer Care and Survival Service (POCSS) collects detailed site-specific and treatment-related data to facilitate outcome evaluation, and TMC has contributed to strengthening national cancer surveillance through its affiliated registries. The case abstraction process has evolved substantially over time, with manual review of hardcopy case files from 1985 to 2013, followed by early IT-supported abstraction systems in the 2000s, and the implementation of a fully functional electronic medical record (EMR) in 2013 enabling paperless documentation across clinical services.

Given the rising cancer burden and the need for timely, high-quality registry outputs, there was a clear operational need to optimize abstraction workflows without compromising established data standards (11, 12). In 2021, Onco-Insight software was customized and integrated with the EMR at TMC to enable automated retrieval of predefined structured variables from multiple institutional modules including PABR, CIS, OT, MOIS, and ROIS into a unified abstraction interface for both HBCR and POCSS. Since its inception, 206,484 cases have been abstracted between 2019 and 2024, with partial abstraction from 2022 – 2024, simplifying registry data abstraction despite increasing patient volumes. Importantly, this framework does not rely on machine learning or natural language processing; rather, it leverages structured data fields already captured within institutional systems. By consolidating demographic, diagnostic, staging, treatment, and follow-up information into a single abstraction environment, the system was designed to reduce navigation time, minimize duplicate data entry, and harmonize workflows between registry units while preserving data integrity and auditability (13).

This study was done with an objective to assesses, an EMR-integrated Onco-Insight platform for data completion and data entry turnaround time required for abstraction using diagnosis and treatment data from 2021.

## Methodology

### Study design and setting

This was an ambivalent observational study conducted by the Division of Cancer Care, Hospital Based Cancer Registry (HBCR) and Survival Studies within the Centre for Cancer Epidemiology, at the Advanced Centre for Treatment, Research and Education in Cancer (ACTREC), a constituent unit of the Tata Memorial Centre. The study assessed the *Onco- Insight*, an Electronic Medical Record (EMR)-integrated cancer registry software customized in 2021 to facilitate structured data abstraction from institutional EMR systems and to streamline registry workflows.

### System architecture and data capture framework

Onco-Insight, developed in 2021, is an EMR-integrated cancer registry platform structured into four modules: Demographic, Diagnosis and Staging, Treatment, and Follow-Up (**Supplementary Table 1**).

The demographic module auto-populates patient identifiers and sociodemographic variables through direct mapping of structured EMR registration fields. Manual entry is limited to variables not available in structured format, specifically address type (urban/rural/foreign) and year of residence.

The diagnosis and staging module follow a hybrid abstraction model. Structured data from the CIS module are retrieved for variables such as primary site, secondary site, and histology when a final diagnosis is available, and in its absence these variables are manually abstracted. Method of diagnosis, clinical extent, comorbidities and lifestyle factors are manually abstracted.

The treatment module integrates therapeutic data. Structured treatment events (e.g., surgery, radiotherapy, chemotherapy) are auto extracted and displayed in a grid format. Variables such as treatment intent, sequence and performance status are manually entered to complement structured data.

The follow-up module supports outcome documentation. As follow-up data are not fully structured within the EMR, all variables including vital status and disease status are manually entered by trained registry personnel.

### Platform functionality and data integration

Onco-Insight is a Visual Basic–based application that retrieves structured clinical data from multiple Electronic Medical Record (EMR) modules using Structured Query Language (SQL). When a cancer case number is entered into the software, SQL queries are automatically executed in the background to retrieve all available structured information from the respective EMR modules. The case number serves as the unique identifier linking information across modules for a given patient. Retrieved data are automatically populated into the corresponding software screens, including sociodemographic, diagnosis, treatment, and follow-up sections.

The software applies built-in validation checks based on the Indian Council of Medical Research (ICMR) validation checklist, including site–histology matching, stage–metastasis agreement, gender-specific cancer site validation, site-specific histology checks, treatment–diagnosis consistency, prior treatment compatibility with clinical extent, and mandatory field completion. Cases with incomplete structured information, validation discrepancies, treatment updates during follow-up, or variables not consistently available in structured EMR fields undergo manual review and abstraction. Following review and any necessary modifications by the user, the updated data along with the originally retrieved information are saved within the local HBCR database, while the source hospital EMR modules remain unmodified. All retrieved records are displayed within a unified interface, after which manual verification is performed to ensure completeness, consistency, and overall data quality.

Registry staff undergo regular training for EMR-based data abstraction to ensure uniformity and consistency in data capture. The in-house software is concurrently updated whenever modifications are made to the EMR system, maintaining synchronization between both platforms. In addition, ongoing training sessions are conducted to familiarize staff with newly implemented software features, updates, and workflow enhancements, thereby supporting consistency across abstractors.

**Figure 1:**
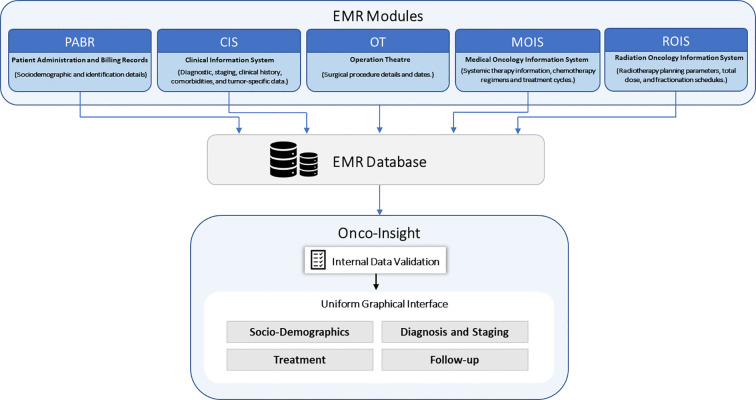
Onco-Insight system architecture.

A schematic workflow is provided in **Figure 1**.

Study procedure:

For objective 1, to evaluate completeness of sociodemographic and diagnostic data fields using 2021 registry data via EMR-integrated software linkage, the data was abstracted for all the cases registered in 2021 and checked how much data was linked with the software. For objective 2, to evaluate completeness of treatment-related data fields using 2021 registry data via EMR-integrated software linkage, the data was abstracted for all the cases registered in 2021 and checked how much data was linked with the software. For objective 3, to evaluate differences in data entry turnaround time between conventional manual abstraction and the Onco-Insight platform, the study was conducted using an intra-observer comparative design to evaluate the data abstraction process. All cancer cases registered at TMC during 2021 with complete EMR records were included in the analysis. The year 2021 was selected because it represents the most recent dataset with complete abstraction following the full implementation of the Onco-Insight platform, whereas abstraction for the years 2022–2024 is still ongoing. A single group of trained registry abstractors performed case abstraction under two conditions: conventional manual abstraction and abstraction using the Onco-Insight platform. A random sample of 100 cases per cancer system was used, incorporating a variety of case types to account for differences in abstraction time. The time required to complete abstraction of a single case was recorded for both approaches. Key variables assessed included sociodemographic details, diagnostic variables (primary site, histology), and treatment-related information. Using available data, the EMR-integrated software populated predefined variables and integrated validation checks, reducing the need for repeated data retrieval and manual verification. Differences in abstraction time between the two approaches were used to assess the impact of the system on workflow efficiency.

## Results

In 2021, Tata Memorial Centre registered 37,495 case files, of which 32,359 (86.30%) were cancer cases eligible for Hospital-Based Cancer Registry (HBCR) abstraction. Following implementation of the Onco-Insight platform, high levels of system-assisted data retrieval were achieved across institutional EMR databases.

Objective 1: To evaluate completeness of sociodemographic and diagnostic data fields using 2021 registry data via EMR-integrated software linkage.

**Figure 2:**
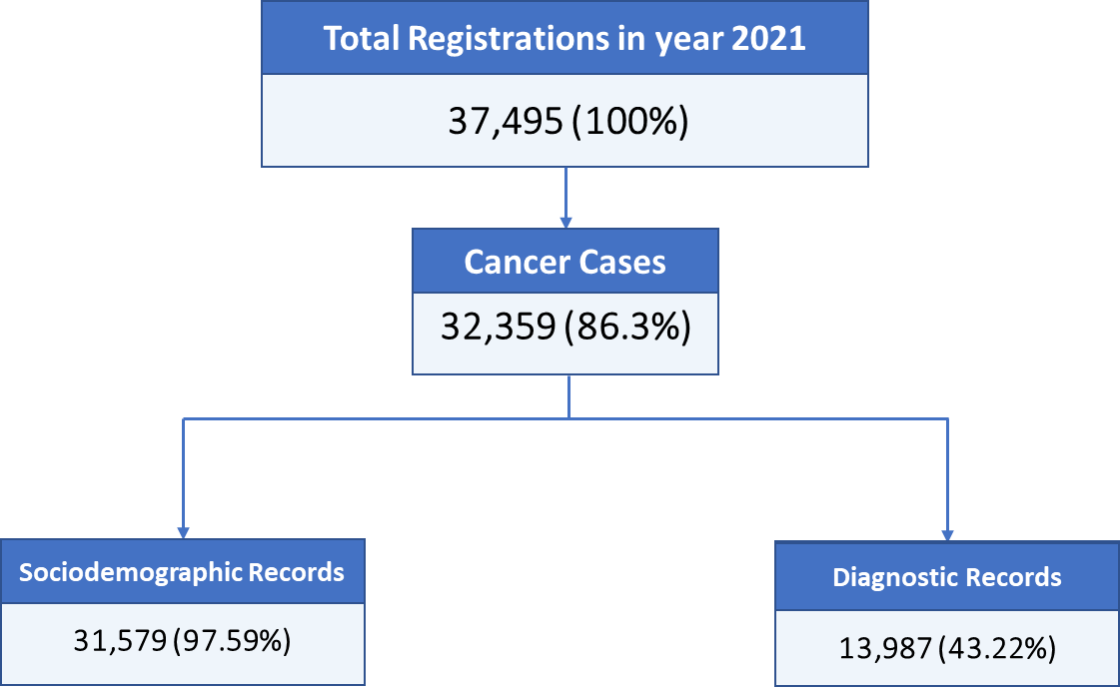
Schematic distribution of cases retrieved for each domain.

The flow chart in **Figure 2** represents the number of cases that were linked with respect to socio- demographic and diagnostic variables. The system-enabled process allowed retrieval of 31,579 (97.59%) sociodemographic records, followed by manual validation. Diagnostic variables like site and histology were retrieved for 13,987 (43.22%) cases, reflecting partial dependence on structured clinical documentation.

**Table 1** presents the number and proportion of cases auto retrieved from the EMR into the software for primary site and histology. Overall, 43.22% of cases were auto retrieved, corresponding to records with an available final diagnosis, whereas 56.78% required manual abstraction due to the absence of a final diagnosis. Site-specific variation was evident: the highest number of linkage was observed for Gastro-intestinal cancers (3318 cases; 49.16%), while the lowest was seen for bone malignancies (281 cases; 46.83%).

**Table 1:** Descriptive comparison of linked versus manually abstracted cases for primary site and histology, stratified by system.

System	Auto retrieved	Manual abstraction	Total
Head & Neck	3184 (43.80)	4085 (56.2)	7269 (22.46)
Breast	2080 (50.57)	2033 (49.43)	4113 (12.71)
Gastro-intestinal	3318 (49.16)	3432 (50.84)	6750 (20.86)
Genito Urinary Cancers	2153 (46.54)	2473 (53.46)	4626 (14.3)
Lung	932 (46.16)	1087 (53.84)	2019 (6.24)
Hematopoietic Cancers	747 (19.76)	3034 (80.24)	3781 (11.68)
Bone	281 (46.83)	319 (53.17)	600 (1.85)
Peripheral Nerves & Soft Tissue	284 (50.09)	283 (49.91)	567 (1.75)
Brain & Nervous System	515 (62.73)	306 (37.27)	821 (2.54)
Others	493 (27.19)	1320 (72.81)	1813 (5.6)
Total	13987 (43.22)	18372 (56.78)	32359 (100)

Objective 2: To evaluate completeness of treatment-related data fields using 2021 registry data via EMR-integrated software linkage.

**Figure 3:**
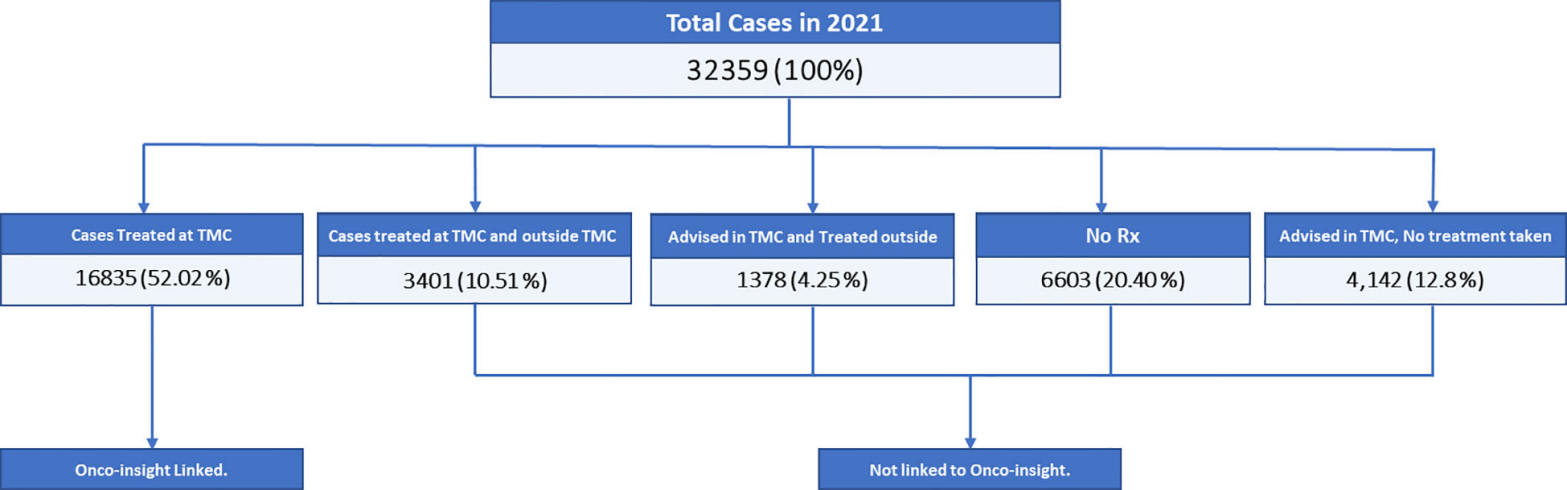
Schematic distribution of cases retrieved for treated cases 2021.

The treatment distribution among 32,359 cases in 2021 and their linkage to the Onco-Insight system. Overall, 16,835 patients (52.0%) received complete treatment at TMC and were fully linked to the Onco-Insight platform, enabling direct EMR-based data capture. In contrast, the remaining categories were not linked to Onco-Insight and required manual abstraction. These included 3,401 patients (10.51%) who underwent treatment both at TMC and outside, 1,378 (4.25%) who were advised treatment at TMC but treated elsewhere, and 6,603 (20.40%) with no treatment (No Rx) recorded. Additionally, 4,142 patients (12.8%) were advised treatment at TMC but did not undergo treatment there. (**Figure 3**)

To assess retrieval accuracy, automatically populated variables were compared against manually abstracted registry records. Registration-related variables demonstrated 97.59% accuracy, reflecting reliable retrieval of structured demographic and identification information directly entered into the EMR. Treatment-related variables demonstrated an overall accuracy of 69%. Treatment data within the software are displayed in a grid format, facilitating efficient manual review and data entry. System-linked treatment records represented cases in which the cancer-directed treatment (CDT) was directly available within the treatment grid through automated EMR linkage, whereas unmatched records primarily reflected additional CDT identified during follow-up, including treatment administered for recurrence or disease progression that was not captured within the initial linked treatment data. Although a substantial proportion of patients had successfully linked treatment data, manual abstraction remained necessary for follow-up treatment events and therapies delivered outside Tata Memorial Centre managed care pathways. (**Table 2**)

**Table 2:** Validation of system-linked cancer-directed treatment (CDT) data against manually abstracted treatment records stratified by treatment received.

Type of treatment received	Auto - retrieved		Total treated
	Matched	Unmatched	
Only Systemic Therapy	6893 (85.92)	1130 (14.08)	8023
R + ST	2323 (71.81)	912 (28.19)	3235
only Radiotherapy	648 (61.19)	411 (38.31)	1059
ONLY Surgery	1411 (64.43)	779 (35.57)	2190
S + ST	1896 (65.00)	1021 (35.00)	2917
S + RT	315 (27.66)	824 (72.34)	1139
S + RT + ST	1457 (47.75)	1594 (52.25)	3051

Treatment and follow up related variables require manual date wise data entry, thus making it difficult to retrieve the data directly from the EMR database.

Objective 3: To evaluate differences in data entry turnaround time between conventional manual abstraction and abstraction using Onco-Insight platform.

**Table 3** compares abstraction time across different cancer sites using manual abstraction and the Onco-Insight system. A consistent reduction in abstraction time was observed with Onco-Insight across most cancer sites, particularly during routine abstraction activities. The greatest reduction was observed for gastrointestinal cancers, where the average abstraction time decreased from 34 minutes 14 seconds using manual methods to 12 minutes 35 seconds using the software. Substantial reductions were also noted for head and neck, lung, and breast cancer cases. Overall, the mean abstraction time decreased from 29 minutes 11 seconds with manual abstraction to 17 minutes 12 seconds with the Onco-Insight system, indicating improved efficiency with system-assisted abstraction across the majority of cancer sites.

**Table 3:** Comparison of time required for cancer case abstraction using manual and EMR-integrated approaches at Tata Memorial Centre for each system.

Sr no.	Systems	Manual abstraction	Abstraction using onco-Insight software
1	Head and Neck	33.03	12.13
2	Gastrointestinal	34.14	12.35
3	Cervix	23.11	10.35
4	Gynecological	19.31	13.21
5	Breast	25.00	15.21
6	Soft tissue	29.18	18.04
7	Bone	30.19	20.52
8	Hemato-Lymphoid	27.19	18.38
9	Brain	29.42	19.42
10	Lung	42.57	26.23
11	Uro- Oncology	27.42	18.11
	Average	29.11	17.12

A paired t-test demonstrated that the mean abstraction time for manual abstraction (29.14 ± 6.15 minutes) was significantly higher than that for abstraction using the Onco-Insight software (16.72 ± 5.03 minutes), with a mean difference of 12.42 minutes (95% CI: 8.99–16.24), t(10) = 8.91, p < 0.001.

## Discussion

This study presents an assessment of a structured EMR-integrated registry platform and its impact on data capture and abstraction efficiency in a high-volume tertiary cancer center. Implementation of the platform was associated with improved data capture and greater efficiency in the abstraction process. Automated retrieval from structured EMR fields enabled complete capture of sociodemographic variables and moderate retrieval of treatment-related data. These findings suggest that structured data within oncology EMRs can improve registry completeness while reducing the need for manual abstraction.

Cancer registries emphasize on completeness, accuracy, comparability and timeliness as core quality dimensions (5–7). Manual abstraction across fragmented hospital systems threatens these dimensions by increasing transcription errors, inter-abstractor variability, and reporting delays (5–7, 14). By centralizing structured data retrieval and embedding validation checks within a unified interface, our system directly addressed these operational vulnerabilities. Importantly, the observed reduction in abstraction time supports improvements not only in efficiency but also in timeliness which is an often under-reported yet critical indicator of registry performance (5).

Our findings align with international efforts to modernize cancer registry infrastructure through EMR integration. In the United States, the Surveillance, Epidemiology, and End Results Program has progressively incorporated electronic reporting standards and automated data exchange mechanisms to improve completeness and timeliness of cancer data (15, 16). Registry–EHR linkage initiatives such as those associated with CancerLinQ have demonstrated that structured integration can enhance treatment data completeness and reduce reporting lag compared with registry-only datasets (11, 13, 15). However, many previously reported systems rely on complex interoperability frameworks or probabilistic linkage methods, which may require substantial technical infrastructure and maintenance (11, 12). In contrast, our approach leveraged existing structured database fields within institutional EMR modules, providing a pragmatic solution in a resource-constrained, high-volume environment.

Within this framework, diagnostic variables achieved a 43.22% linkage rate, obtaining information about primary site and histology where the final diagnosis was available. Thus, highlighting the continued dependence on manual abstraction for complex pathology and interpretative data elements.

Variables related to treatment and follow-up demonstrated comparatively lower automated retrieval rates. These variables often require longitudinal, date-wise manual clinical documentation across multiple encounters, making direct extraction from the EMR database more challenging. In several instances, treatment sequencing, follow-up status, and interim clinical updates were recorded as manually entered entries rather than consistently mapped structured fields, thereby limiting complete automated linkage. In addition, clinical extent was not always explicitly documented in structured EMR fields and often required interpretation from clinical notes, necessitating manual abstraction. Consequently, selective manual verification and abstraction remained necessary for these variables.

Prior studies have demonstrated that natural language processing (NLP) and machine learning–based approaches can improve extraction of complex clinical information from unstructured free-text records (16, 17). However, implementation of these approaches often requires large annotated datasets, repeated model training and validation, substantial computational infrastructure, and specialized technical expertise (4, 18). In addition, AI-based extraction methods may introduce challenges related to reproducibility, auditability, explainability, and variability in performance across institutions and documentation practices.

In the present setting, a substantial proportion of the required registry variables were already available as structured fields within the institutional EMR and were directly entered by treating clinicians during routine clinical care. Consequently, the use of structured-field extraction enabled retrieval of standardized data at the source level, thereby reducing dependence on interpretation of free-text clinical narratives and lowering the likelihood of abstraction-related errors. Since the EMR documentation was maintained in English using predefined structured formats, language-processing limitations commonly associated with NLP-based systems were minimized. Compared with AI- or NLP-based approaches, the structured-data extraction model also offered lower implementation cost, transparent rule-based validation, easier reproducibility, greater auditability, and reduced computational requirements, making it particularly feasible for large-scale hospital-based cancer registries in resource-constrained settings. Our findings demonstrate that substantial improvements in efficiency and data completeness can be achieved when key demographic, diagnostic, and treatment variables are systematically captured within EMR systems.

The average time for manual abstraction was 29 minutes and 11 seconds and with the help of software it has reduced to 17 minutes and 12 seconds. It was seen that without the intervention of software, more time was taken for abstraction compared to the abstraction after linking with EMR. When compared with other registry studies, the time taken for manual abstraction was more compared to that with any integrated software or use of AI.

When compared with international registry modernization initiatives, our experience underscores the importance of workflow redesign rather than technology alone. A comparable trajectory is observed in China, where the National Central Cancer Registry of China has expanded structured hospital-based reporting networks to strengthen national cancer surveillance (19, 20). Although digital reporting has improved case ascertainment and timeliness in several urban tertiary centers, heterogeneity in EMR standardization and structured documentation practices persists across regions (20). Hybrid workflows combining electronic retrieval with manual abstraction remain common where health information systems vary in maturity.

Studies evaluating registry automation in North America and Europe report improvements in abstraction efficiency but also emphasize challenges related to interoperability standards, data mapping, and governance (11, 13, 15, 21). In contrast, the integration achieved in our setting was facilitated by institutional control over EMR and standardized data entry practices within oncology services.

Importantly, registry modernization must remain aligned with established methodological standards. In India, registry operations under the National Cancer Registry Programme, coordinated by the Indian Council of Medical Research through the National Centre for Disease Informatics and Research, emphasize standardized coding practices (ICD-O), completeness of case ascertainment, uniform staging documentation, timeliness of reporting, and systematic quality control (18, 22). Registry software systems should therefore enhance efficiency while preserving comparability, auditability, and adherence to national data standards.

Within this framework, an ideal oncology registry software architecture, particularly in LMIC settings should incorporate: (i) structured data capture at the point of care using standardized templates; (ii) deterministic automated extraction of predefined registry variables; (iii) embedded validation rules consistent with registry quality control protocols; (iv) interoperability with national reporting formats; and (v) governance safeguards including audit trails and controlled access (5, 18, 22). Selective augmentation with NLP or AI-based tools may be appropriate for complex narrative fields, although structured-field optimization remains foundational.

The current software–EMR integration was developed specifically for the Tata Memorial Centre architecture, where EMR modules, variable structures, and database organization are institution-specific. Consequently, direct implementation in other centers may require customization of query logic, variable mapping, and integration workflows according to the local EMR configuration and registry requirements. Nevertheless, the overall framework of structured-field extraction linked to registry abstraction workflows may be adaptable to other hospital-based registries, provided that key clinical variables are systematically captured within structured EMR fields and appropriate technical infrastructure and coordination between clinical, registry, and information technology teams are available. Future integration with interoperability standards such as HL7/FHIR may further improve portability and scalability across different EMR systems.

This study demonstrates key strengths in the implementation of an EMR-integrated registry platform within a high-volume tertiary cancer setting. Integration with existing EMR modules enabled standardized and reproducible data abstraction, reducing variability associated with manual processes. The use of structured data fields ensured high completeness for core variables, particularly sociodemographic and treatment data, while embedded validation checks improved internal consistency. The reduction in abstraction time indicates enhanced efficiency and timeliness of registry workflows. These improvements support the availability of more complete and timely data, strengthening their use for policy planning.

Despite these improvements, some limitations remain. There is still a delay in availability of complete registry data, mainly due to the need for manual review and limited manpower. Although the system retrieves structured data, manual abstraction is still required for unstructured and follow-up variables, which increases workload. The platform also does not include AI-based tools, limiting automatic extraction of information from free-text clinical records. In addition, lack of consistent structured data entry by clinicians reduces the extent of automated data retrieval. Addressing these issues will require better structured documentation, improved staffing, and selective use of AI tools to further improve efficiency and timeliness.

## Conclusion

Overall, this study shows that integrating electronic medical records into registry workflows can meaningfully improve data completeness and make the abstraction process faster and more consistent in a high-volume cancer center. By reducing reliance on manual effort and enabling more standardized data capture, such systems also support more timely and reliable registry data. However, their effectiveness depends on how well data are structured at the point of care and on continued refinement of extraction processes. In high-burden settings, these improvements can play an important role in strengthening cancer registries and supporting better-informed clinical research and cancer control planning.

## Data Availability

The original contributions presented in the study are included in the article/**Supplementary Material**. Further inquiries can be directed to the corresponding author.

## Supplementary material

Supplementary Table 1 The Essential variables that are abstracted for HBCR data entry from the EMR.
