# Supplementary material for: “Assessment of an EMR-integrated Onco-Insight hospital-based cancer registry system for data completeness and data entry turnaround time at a tertiary care cancer center in India”
Source: Front Oncol. 2026 Jun 9;16:1852225. doi: 10.3389/fonc.2026.1852225 (PMC13286784; doi:10.3389/fonc.2026.1852225)
Supplement: Supplementary Table 1 — The Essential variables that are abstracted for HBCR data entry from the EMR. [file Table1.docx]

**Supplementary tables**

**ST1: The Essential variables that are abstracted for HBCR data entry from the EMR**

| **Essential Variables** | |
| --- | --- |
| 1. **Sociodemographic Screen**   **Identification**   - - CASENO   - CANCN   - Name   - Registration Date   - Diagnosis Date   - Type of Case   **Next of Kin**   - - Name of Kin   - Relationship   **Address Details**   - - Address Type   - Permanent Address   - PPIN   - Local Address   - LPIN   **Contact Information**   - - Office Phone   - Residence Phone   - Mobile No. 1   - Mobile No. 2   - E-mail ID   **Identification**   - - Aadhar Number   **Referral Details** | 1. **Treatment Screen**   **Prior Treatment Details**   - - Treatment Given Prior to Registration at RI   - Type of Treatment Given   - Date of Prior Treatment Start   - Prior Surgery Date   - Prior RT Completion Date   - Prior Chemotherapy Completion Date   - Hormone Start Date   - Teletherapy Dose (Prior)   - Brachytherapy Dose (Prior)   - Other Dose (Prior)   - Chemo (Prior)   - Hormone Therapy (Prior)   **Intention to Treat**   - - Intention to Treat   - Whether Treatment Taken as Planned   - Type of Treatment Advised but Not Taken   **Current Treatment Details**   - - Type of Treatment Received as per TMC Advised   - Sequence of Treatment   - Date of Treatment Start   - Date of Surgery   - RT Start Date   - RT End Date   - CT Start Date |

| **Essential Variables** | |
| --- | --- |
| - Name of Referring Doctor - Address of Referring Doctor   **Personal Details**   - Date of Birth - Age - Sex - Duration of Stay (in years)   **Clinical Background**   - Serology – HIV - Serology – HBsAg - Serology – HCV   **Socioeconomic Details**   - Education - Marital Status - Occupation - Mother Tongue - Religion - Family Income  1. **Diagnosis and Staging Screen**   **Diagnostic Information**   - - Diagnosis   - Diagnostic Status at RI   - Method of Diagnosis   **Tumor Details**   - - Primary Site of Tumour   - ICD-10   - ICD-9   - Primary Histology   - P-Grade   - Secondary Site of Tumour   - Secondary Site Histology   - S-Grade   **Tumor Characteristics**   - - Laterality   - Sequence   **Registry Information**   - - PF Number   **Report Details Table**   - - Sr No   - Report Date   - Modality   - Centre   - Impression   - Site   - Histology   - Addendum   **Staging**   - - Clinical Extent of Disease | - CT End Date - HT Date - Hormone Therapy Type - Hormone Others - Teletherapy - Brachytherapy - Others (RT Type)   **Institutional Details**   - Treatment Received at TMC - Treatment Advised at TMC and Received Outside   **Performance Status**   - Performance Status Before Treatment - Performance Status at 6–12 Weeks After Completion of CDT - Date of Assessment of Performance Status   **Outcome at Discharge**   - Disease Status at Discharge   **Treatment Delay**   - Treatment Delay Reason   **Complications**   - Click to Enter Complications During Treatment   **Mortality**   - Patient Died (Yes/No) - Death Date - Cause of Death   **Registry Information**   - Second Primary - TMH Remarks - Entered By   **Treatment Report Table**   - SR No - CASENO - RX_TYPE - RX_DATE - INST - DETAILS1 - DETAILS2 - DETAILS3   **Site-Specific Modules**   - Head & Neck - Breast - Cervix - Haematolymphoid - Others - Other Gynae  1. **Follow up Screen**   **Follow-Up Details**   - - FUP No   - Date of FUP   - Method of FUP   - Vital Status |

| **Essential Variables** | |
| --- | --- |
| - T (Tumor) - N (Nodes) - M (Metastasis) - Stage   **Staging (POCSS)**   - Disease Status at Diagnosis (Haematolymphoid)   **Additional Modules**   - Co-morbidity / Lifestyle details - Stage Details - Breast - Head & Neck - Cervix - Other Gynae - Haematolymphoid - OESO - Select Other Sites | - Disease Status - Basis of Diagnosis / Justification - Metastatic Site - Treatment Received - Type of Recurrence During FUP - Late Complications - Demography/Comorbidity/Investigation Update - Click for Demo change   **Second Primary Details**   - Second Primary - Basis of Diagnosis - Primary Site - Primary Histology - Metastatic Site - Metastatic Histology - Date of Diagnosis   **Death Details**   - Dead Cause of Death - FUP Remarks |
